# Novel Antibiotics for Multidrug-Resistant Gram-Positive Microorganisms

**DOI:** 10.3390/microorganisms7080270

**Published:** 2019-08-18

**Authors:** Despoina Koulenti, Elena Xu, Isaac Yin Sum Mok, Andrew Song, Drosos E. Karageorgopoulos, Apostolos Armaganidis, Jeffrey Lipman, Sotirios Tsiodras

**Affiliations:** 1 UQ Centre for Clinical Research, Faculty of Medicine, The University of Queensland, Brisbane, QLD 4072, Australia; 2 2nd Critical Care Department, Attikon University Hospital, 12462 Athens, Greece; 3 4th Department of Internal Medicine, Attikon University Hospital, 12462 Athens, Greece; 4 Department of Intensive Care Medicine, Royal Brisbane and Women’s Hospital, Brisbane 4029, Australia; 5 Anesthesiology and Critical Care, Centre Hospitalier Universitaire De Nîmes (CHU), University of Montpellier, 30029 Nîmes, France

**Keywords:** multidrug-resistance, gram-positive pathogens, novel antibiotics, ceftobiprole, ceftaroline, telavancin, oritavancin, dalbavancin, tedizolid, besifloxacin, delafloxacin, ozenoxacin, omadacycline

## Abstract

Increasing multidrug-resistance to Gram-positive pathogens, particularly to staphylococci, enterococci and streptococci, is a major problem, resulting in significant morbidity, mortality and healthcare costs. In recent years, only a small number of novel antibiotics effective against Gram-positive bacteria has been approved. This review will discuss the current evidence for novel branded antibiotics that are highly effective in the treatment of multidrug-resistant infections by Gram-positive pathogens, namely ceftobiprole, ceftaroline, telavancin, oritavancin, dalbavancin, tedizolid, besifloxacin, delafloxacin, ozenoxacin, and omadacycline. The mechanism of action, pharmacokinetics, microbiological spectrum, efficacy and safety profile will be concisely presented. As for any emerging antibiotic agent, resistance is likely to develop against these highly effective antibiotics. Only through appropriate dosing, utilization and careful resistance development monitoring will these novel antibiotics continue to treat Gram-positive pathogens in the future.

## 1. Introduction

Antibiotic resistance is increasingly becoming a global health threat associated with increased morbidity, mortality, prolonged hospitalization and healthcare costs [1]. It is estimated that 300 million people will die prematurely from drug resistance over the next 35 years if antibiotic resistance is not overcome [2]. The above additionally translates into a $USD 60 to 100 trillion worth of economic output lost by 2050 [2]. Over the past five years, a number of antimicrobial agents that are effective against Gram-positive multidrug-resistant organisms have been developed and approved as a result of clinical trials [3]. Most of these registrational clinical trials are done on community-acquired infections requiring hospitalization, mainly on community-acquired pneumonia and on skin and soft tissue infections. However, the newer agents could also play a role in the treatment of nosocomial infections, when resistance, toxicity, drug interactions or treatment failure precludes treatment with established agents.

Bacterial strains with non-susceptibility to one or more antimicrobials in three or more antimicrobial classes, are defined as multidrug-resistant (MDR) [4]. These MDR strains have been increasing at an alarming rate over recent decades, making it more difficult, sometimes impossible, to treat common bacterial infections with conventional antibiotics [5].

*Staphylococcus aureus*, the most common Gram-positive MDR pathogen causing nosocomial infections, is a major cause of morbidity and mortality worldwide [6]. It is a frequent cause of both hospital-acquired and community-acquired infections in both healthy individuals and patients with risk factors or underlying conditions [6]. *S. aureus* can cause a wide range of infections, from mild skin infections to life-threatening diseases, including pneumonia, osteomyelitis, sepsis and bacteremia [7]. Methicillin-resistant *S. aureus* (MRSA) is considered a serious threat by the Centre for Disease Control and Prevention (CDC) and classified as high priority by the Public Health Agency of Canada (PHAC) [6]. Several studies reported that patients infected with MRSA have an elevated 30- and 90-day risk of mortality and a 1.19-fold increase in hospital charges compared to those with methicillin-susceptible *S. aureus* (MSSA) [8,9].

Another Gram-positive pathogen, *Enterococcus faecium*, is of particular concern due to its potential resistance to nearly all antimicrobials currently used in medical practice [10]. It has a high propensity to persist in the hospital environment owing to its ability to adapt to the harsh environment of the gastrointestinal tract and flourish under antibiotic pressure [6]. As a result, it is a frequent cause of hospital-acquired infections, such as bloodstream and urinary tract infections, which can be detrimental in critically ill and immunocompromised patients [6]. Vancomycin-resistant *E. faecium* has been classified as a serious threat by the CDC and medium-high priority by PHAC [6].

*Streptococcus pneumoniae* is responsible for several community-acquired infections, including pneumonia, meningitis, acute exacerbations of chronic obstructive pulmonary disease (COPD) and sinusitis [11]. Studies have shown certain strains of *S. pneumoniae* to be resistant to penicillin, clindamycin, cotrimoxazole and erythromycin, rendering these treatment options futile [11]. CDC classified penicillin-resistant *S. pneumoniae* as a serious threat, whilst the PHAC classifies it as medium-low priority [6].

This review will discuss antibiotics against Gram-positive micro-organisms approved during the last decade, including the mechanism of action and microbiological profile as demonstrated in in vitro and in vivo experiments, as well as dosing, safety and efficacy of the antibiotics demonstrated in clinical trials. Targeted searches included PubMed, Google Scholar, official label information, the site of ClinicalTrials.gov, and recent conference proceedings (of: Infectious Diseases Society of America, European Society of Clinical Microbiology and Infectious Diseases, American Society of Microbiology). (For novel and emerging antibiotics against Gram-negative antibiotics, we refer to another recent review [12]).

Table 1 summarizes the novel anti-Gram-positive antibiotics approved during the last decade. (Chemical structures summarized in Supplementary Material).

## 2. Novel Anti-Gram-Positive Antibiotics

### 2.1. β-Lactams

#### 2.1.1. Ceftaroline

Ceftaroline (trade name Zinforo under a license from Pfizer except for US, Canada and Japan; trade name Teflaro under license by Allergan Pharmaceutical Industries Limited in US/Canada and by Takeda Pharmaceutical Company Limited in Japan) is a novel 5th generation cephalosporin that was approved by the Food and Drug Administration (FDA) in 2010 and from the European Medicine Agency (EMA) in 2012 for the treatment of acute bacterial skin and skin structure infections (ABSSSIs) and community-acquired bacterial pneumonia (CABP) [26]. Its mechanism of action involves cell wall synthesis inhibition by inactivating penicillin-binding proteins (PBP), and it binds to PBP-1a, -2a, -2b, and -2x proteins with a greater affinity than other penicillin and cephalosporin antibacterial drugs, giving it enhanced activity towards MRSA and penicillin-resistant *S. pneumoniae* [27,28]. Ceftaroline has bactericidal activity against a wide spectrum of Gram-positive aerobes, including MSSA, MRSA, vancomycin-resistant *S. aureus*, daptomycin non-susceptible *S. aureus*, linezolid-resistant *S. aureus*, *Streptococcus pyogenes*, *Streptococcus agalactiae*, multiple strains of resistant *S. pneumoniae,* while the activity against *Enterococcus faecalis* (including vancomycin-resistant enterococci [VRE] strains) is modest (not active against *E. faecium*) [29,30]. The antimicrobial spectrum of ceftaroline against Gram-negative bacteria is similar to that of ceftriaxone [31].

Ceftaroline is administered by intravenous (IV) injection in a water-soluble prodrug formulation, ceftaroline fosamil, which then undergoes in vivo conversion by plasma phosphatases almost immediately [32]. It has a low plasma protein binding (average 20%) and a terminal half-life of 1.6 and 2.6 h after a single dose and after multiple doses, respectively [33]. Pharmacokinetic studies of ceftaroline epithelial lining fluid (ELF) distribution among healthy adult subjects demonstrated rapid penetration into ELF, with maximal concentrations above minimum inhibitory concentration (MIC_90_) of MRSA when given every 8 or 12 h [34]. The currently approved dosing regimen in adults with CABP and ABSSSI are 600 mg every 12 h by IV infusion over one hour for five to seven days and five to 14 days, respectively [13,35]. However, based on evidence of PK/PD changes of other beta-lactam antibiotics in critically ill patients, 600 mg every 8 h should be considered as it is safe and is expected to be a better dosing regimen for critically ill patients with normal renal function (normal or augmented renal clearance) [23]. The pharmacokinetics of ceftaroline in pediatric patients appear similar to those of adults; the dosing recommendations for pediatric patients are weight-based (8 mg/kg every 8 h for 2 months to younger than 2 years; 12 mg/kg every 8 h for 2 years to younger than 18 years weighing up to 33 kg) [33]. Dosage adjustment is required in renal impairment, specifically for patients with an estimated creatinine clearance at 50 mL/min or less [32]. Dose adjustments are made by reducing the dose instead of the dosing interval, as the efficacy of ceftaroline is mainly determined by free-drug concentration above MIC [36]. The commonest adverse drug events are diarrhea, nausea, and rash, with vomiting, headache, hypokalaemia, increased liver transaminases, and phlebitis also documented (2–3% among patients from four phase III clinical trials) [37]. Other notable adverse events with incidence rates below 2% are bradycardia, palpitations, pyrexia, *Clostridium difficile* colitis, anemia, eosinophilia, neutropenia, thrombocytopenia, and neutropenia [33]. Regarding neutropenia, it is noteworthy that a retrospective study from US reported increased incidence after prolonged ceftaroline exposure, namely 10–14% for ≥2 weeks and 21% ≥3 weeks of exposure, indicating the need for close laboratory monitoring in cases of long-term administration [38].

A randomized-controlled phase III trial in adult patients with complicated skin and soft tissue infections (cSSTIs) found that high-dose ceftaroline fosamil at 600 mg every eight hours was non-inferior in clinical cure rates compared to vancomycin plus aztreonam (NCT01499277) [39]. It should be mentioned that ceftaroline has not been evaluated for MRSA pneumonia in clinical trials. The relevant registrational studies for CABP (FOCUS 1 & 2) included ceftriaxone as the comparator, which lacks activity against MRSA [40]. The overall number of cases of *S. aureus* CABP was relatively small; ceftaroline was effective in 18 out of 25 (72%) such cases, while ceftriaxone was effective in 15 out of 27 (55.6%), respectively [40]. The number of MDR *S. pneumoniae* cases was inadequate to allow for safe conclusions [40].

An emerging concern with ceftaroline use is the reported decreased susceptibility and increased resistance in certain MRSA clinical isolates collected around the world [36,41,42,43]; especially high regional resistance percentages have been reported from China, Thailand, and Australia [36,43]. In particular, the in vitro study that evaluated MRSA isolates from Melbourne, Australia found significant ceftaroline resistance among MDR phenotype and the CC239 strain making it a non-ideal option for empiric treatment in suspected or known MRSA infections in regions where strain CC239 represents a significant proportion of MRSA [36]. It should be noted however, that most resistance strains have been re-categorized as intermediate with the updated The European Committee on Antimicrobial Susceptibility Testing (EUCAST) breakpoints [44].

A recent meta-analysis of 10 randomized control trials (RCTs) that compared the safety and efficacy of ceftaroline versus comparators, reported a similar rate of adverse events and a significantly lower treatment failure for ceftaroline, with pooled risk ratio (RR) 0.79 (95% CI 0.65–0.95) [45]. It should be noted, however, that no RCTs were identified for non-complicated cSSSIs or for pneumonia other than of CAP, that the number of cases with MRSA were few, and that there were no ICU patients included [45]. Another recent meta-analysis that compared ceftaroline with ceftriaxone for CABP found that both clinical efficacy and safety were similar between the two groups, while a meta-analysis that focused on cSSSIs concluded that ceftriaxone and comparators had similar cure rates, microbiological eradication rates and similar safety profile [46].

Ceftaroline’s spectrum of activity, efficacy and safety profile makes it a promising and attractive addition to the available agents for the treatment of patients with sSSSIs and CABP, providing the option of monotherapy. However, further studies are needed to assess the effectiveness of ceftaroline in the ICU setting and for indications other than CABP and cSSSIs.

#### 2.1.2. Ceftobiprole

Ceftobiprole (trade name Zevtera/Mabelio) is a novel 5th generation cephalosporin developed by Basilea Pharmaceuticals. It exerts its activity by binding to PBPs, including the PBP-2a of MRSA, and blocking bacterial cell wall synthesis [47]. It has a potent activity against a broad spectrum of microorganisms that includes Gram-positives, such as MRSA, ampicillin-susceptible enterococci, penicillin-resistant pneumococci and Gram-negatives; its Gram-negative spectrum is similar to that of 3rd and 4th generation cephalosporins, i.e., not active against Gram-negative strains producing extended-spectrum beta lactamases [47,48].

Ceftobiprole has a very low plasma protein binding (average 16%), has a half-life of approximately 3 h and is eliminated predominantly by the kidneys so dose adjustments are required for patients with moderate to severe renal impairments [14]. In healthy volunteers, ceftobiprole demonstrated mean and median ELF penetration percentages of 25.5% and 15.3%, respectively [49].

In two earlier randomized, multi-center, double-blind phase III trials (STRAUSS I and II) evaluating the clinical efficacy of ceftobiprole for hospitalized patients with complicated skin and skin structure infections (cSSSIs), ceftobiprole demonstrated non-inferiority in clinical cure rates at the test of cure (TOC) visit compared with vancomycin monotherapy and vancomycin/ceftazidime in STRAUSS I and II trials, respectively [50]. More recently, in a phase III, randomized, double-blind, comparative (NCT00326287) study comparing ceftobiprole medocaril to ceftriaxone/linezolid for CABP, ceftobiprole achieved a cure rate of 76.4% compared with that of 79.3% for ceftriaxone/linezolid [95% CI: −9.3% to 3.6%] while both drugs being well tolerated [51]. In another randomized double-blind phase III trial (NCT00210964/NCT00229008), ceftobiprole medocaril was compared to ceftazidime/linezolid for hospital-acquired pneumonia (HAP) including ventilator-associated pneumonia (VAP) [52]. The cure rate for ceftobiprole was 49.9% compared to that of 52.8% in ceftazidime/linezolid [95% CI: −10% to 4.1%] with similar adverse event rates at 24.9% and 25.4% respectively, displaying non-inferiority [52]. However, in VAP patients, ceftobiprole did not demonstrate non-inferiority in terms of both the cure rate and microbiological eradication rate compared with ceftazidime/linezolid [52].

The most commonly (≥3% of patients) reported adverse reactions of ceftobiprole were nausea (4%), diarrhea (4.2%), neutropenia (4.0%) and drug hypersensitivity reactions including urticaria and pruritic rash (3.0–9.3%) [14,53,54].

Recently a phase III non-inferiority clinical trial (TARGET study, NCT03137173) that compared ceftobiprole with vancomycin plus aztreonam for ABSSSI, was completed [55]. On 6^th^ of August 2019 positive topline results were announced: the primary endpoint that was early clinical response was met (91.3% for ceftobiprole versus 88.1% for vancomycin plus aztreonam) [55]. The two secondary endpoints of clinical success at TOC visits (days 15 to 22) were met as well, for both the intention-to-treat (ITT) population (90.1% versus 89%) and the clinically evaluable population (97.9% versus 95.2%) [56]. Ceftobiprole was tolerated well and the adverse events were similar between ceftobiprole and comparator (20% versus 18%, respectively) [56].

Ceftobiprole was approved by EMA in October 2013 for the indications of HAP (excluding VAP) and CABP [56]. Treatment may continue in patients with HAP who subsequently deteriorate and require mechanical ventilation. It is marketed in many European countries as well as several other countries, such as Canada and Argentina [56]. Ceftobiprole has also received Qualified Infectious Disease Product (QIDP) designation from the FDA for *S. aureus* bacteremia (SAB), ABSSSI and CABP in the United States [56]. A phase III trial evaluating the safety and efficacy of ceftobiprole medocaril compared with daptomycin in treating SAB, including infective endocarditis, is currently recruiting and is expected to be completed in August 2021 (ERADICATE study, NCT03138733) [57]. ERADICATE study along with TARGET study are both under a Special Protocol Agreement with FDA [56].

Ceftobiprole represents an effective and well-tolerated addition to the antimicrobial armamentarium for the empirical treatment of HAP patients (but not for VAP patients). Due to its broad-spectrum of activity, it provides a promising monotherapy alternative to the combination therapy. However, more studies are needed to further assess the activity of ceftobiprole in MRSA HAP against comparators, i.e., linezolid or vancomycin.

### 2.2. Glycopeptides

#### 2.2.1. Dalbavancin

Dalbavancin (trade name Dalvance in US, Xydalba in Europe), developed by Durata Therapeutics (acquired by Actavis in 2014), is a semisynthetic lipoglycopeptide. Its mechanism of action is similar to vancomycin, which consists of binding to D-alanyl-D-alanine terminus of the stem pentapeptide in peptidoglycan, thereby preventing cross-linking of cell wall synthesis [58]. However, dalbavancin has an additional lipophilic side chain that anchors it to the cellular membrane, allowing it to have enhanced activity and four to eight times the potency of vancomycin [58,59]. Dalbavancin’s in vitro spectrum of antibacterial activity covers MRSA, *S. pyogenes*, *S. agalactiae*, *Streptococcus anginosus* group (including *S. anginosus, S. intermedius*, and *S. constellatus*), and *E. faecalis* strains susceptible to vancomycin [60]. It is important to note enterococcal species with Van-A phenotype expression have resistance against dalbavancin [58].

The half-life of dalbavancin is approximately 8.5 days, allowing it to be administered once-weekly in a two-dose regimen [59,61,62,63]. It has high plasma protein binding (average 93%; mainly albumin) that is concentration independent and not affected by renal or hepatic insufficiency [16]. Dalbavancin’s mean plasma clearance decreases and area under the curve increases in patients with creatinine clearance below 30 mL/min, requiring dosage reduction [64]. It is administered by IV infusion over 30 min [60].

DISCOVER I and DISCOVER II are two identically designed, randomized, double-blind, double-dummy phase III trials comparing the efficacy and safety of dalbavancin to vancomycin and linezolid for the treatment of ABSSSIs (NCT01339091 and NCT01431339), and both studies demonstrated that IV dalbavancin 1000 mg on day 1 and 500 mg on day 8 is non-inferior to IV vancomycin (1 g every 12 h or 15 mg/kg every 12 h) with optional switch to oral linezolid (600 mg every 12 h) for 10–14 days [61]. The three commonest adverse effects from both treatment groups of dalbavancin and vancomycin-linezolid were nausea, diarrhea, and pruritus [61]. Infusion site-related reactions and flushing occurred less frequently in the dalbavancin group compared to the vancomycin-linezolid group (1.4% vs. 1.7% and 0.2% vs. 0.6%, respectively) [61]. A randomized, double-blind phase III trial (NCT02127970) in patients with ABSSSIs found that a single dose of dalbavancin 1500 mg by IV infusion is non-inferior to the two-dose regimen in reducing >20% of erythematous area at 48–72 h (81.4% vs. 84.2%; difference, −2.9% [95% CI, −8.5% to 2.8%]), as well as non-inferior clinical outcomes on day 14 and day 28 [65]. The frequency of treatment-emergent adverse events was similar in both groups [65].

In 2014, dalbavancin was approved for treating ABSSSIs in adults by both the FDA and the EMA [16,66]. To date, there is one phase III trial currently recruiting participants to compare dalbavancin for treating ABSSSIs in children aged three months to 17 years of age (NCT02814916) [67]. There are currently five phase IV clinical trials (two completed but pending results (NCT02940730 and NCT03233438), two recruiting participants (NCT03426761 and NCT03372941) and one not yet recruiting (NCT03982030)). The two completed studies evaluate the pharmacokinetic properties of dalbavancin in patients undergoing chronic peritoneal dialysis (NCT02940730), the other one entails the comparison of a New Critical Pathway using dalbavancin versus the standard of care in clinical practice for ABSSSIs treatment (NCT03233438) [68,69]. A phase III trial of dalbavancin in CABP sponsored by the manufacturer (NCT02269644) has been withdrawn prior to enrolment [70]. Data regarding the intrapulmonary pharmacokinetics of dalbavancin are scarce [71].

Due to its long half-life and, thus, weekly administration dosing scheme, dalbavancin has a very promising potential to decrease the duration of hospital stay and for use in the Outpatient Parenteral Antibiotic Therapy population, especially for vulnerable and/or patients with poor adherence to medications [72,73].

#### 2.2.2. Oritavancin

Oritavancin (trade name Orbactiv), developed by Melinta Therapeutics, Inc., is a new-generation lipoglycopeptide derived from chloroeremomycin, an analogue of vancomycin [74]. Oritavancin has rapid bactericidal activity against Gram-positive bacteria, including MSSA, MRSA, VRE and vancomycin-intermediate and vancomycin-resistant staphylococci [74]. Unlike its precursors, oritavancin disrupts the bacterial membrane integrity leading to the bactericidal killing of Gram-positive pathogens [75]. It also appears to inhibit bacterial RNA synthesis [74]. The spectrum of activity is similar to vancomycin, but with a lower MIC (MIC_90_ of 0.008 μg/mL against *S. pneumoniae*, MIC_90_ of 0.12 μg/mL against *E. faecium* isolates, and MIC_90_ of 0.2 μg/mL against *S. aureus*) [76]. In vitro, oritavancin has activity against *Enterococcus* isolates with Van-A (MIC: >4 mg/L vancomycin & >8 mg/L teicoplanin) and Van-B ((MIC: >4 mg/L vancomycin & ≤8 mg/L teicoplanin) phenotypes (while the other novel members of the class, i.e., dalbavancin & telavancin, have activity only against Van-B [77].

The unique lipophilic side-chain of oritavancin prolongs the half-life compared to vancomycin (terminal half-life >10 days, the longest of the glycopeptide class), allowing for single-dose treatment [17,78]. Oritavancin has high plasma protein binding (average 80%) and is eliminated from tissue sites at a slow rate, with no dose adjustments required for mild to severe renal or moderate hepatic insufficiency or other subpopulations including age, gender, race and weight [78,79,80].

Two identical, multi-center, randomized non-inferiority phase III trials, SOLO I and SOLO II, compared the efficacy and safety of a single dose of 1200 mg oritavancin IV oritavancin versus vancomycin IV (1 g or 15 mg/kg) twice daily for 7–10 days in adults with ABSSSI due to proven or suspected Gram-positive pathogens [17,81]. The initial protocol required patients to be hospitalized until the early clinical assessment (48–72 h after treatment initiation), the protocol was amended to include the entire treatment at outpatient setting based on the investigator’s discretion [82]. Although efficacy response rates and the incidence of adverse events were similar for oritavancin and vancomycin, oritavancin provides a single-dose alternative to multi-dose vancomycin for the treatment of ABSSSI [17,82]. A post-hoc analysis of the subgroup of patients that received the entire treatment course in an outpatient setting also concluded that oritavancin and vancomycin had similar efficacy [82].

The most commonly reported adverse reactions in pooled ABSSSI clinical trials for oritavancin compared to vancomycin were headache (7.2% vs. 7.9%), nausea (11.0% vs. 8.9%) and vomiting (4.9% vs. 3.7%), diarrhea (4.9% vs. 3.5%), limb and abscess limb (2.8% vs. 1.6%), however treatments were rarely discontinued [17,79,83,84]. The high intracellular accumulation of oritavancin suggests potential toxicity in tissues, such as the liver and lungs, therefore close monitoring is required to determine the side effects until more clinical data is available [83,85].

In August 2014 and March 2015, the FDA and EMA, respectively, approved oritavancin for the treatment of ABSSSI due to MSSA, MRSA, *Streptococcus* spp., and *E. faecalis* in adults administered via IV infusion of 1200 mg single dose over three hours [74,79,80,86]. Despite potent antimicrobial activity, oritavancin has not been clinically evaluated against VRE. A multi-center phase I clinical trial is currently recruiting participants to evaluate the safety and tolerability of IV oritavancin in pediatric patients (<18 years old) with Gram-positive bacterial infections (NCT02134301) [87]. Another phase IV clinical trial, estimated to be completed by the end of 2019, is evaluating the safety and efficacy of oritavancin in adult patients with a systemic infection of *S. aureus*, namely bacteremia or infective endocarditis (NCT03761953) [88].

A single dose of oritavancin due to its long half-life, along with its safety profile, makes it a very promising option for effectively shifting inpatient to outpatient setting care for patients with ABSSSI and, thus, substantially decreasing the related healthcare costs. Future studies are needed, however, to evaluate the role of oritavancin in the treatment of enterococcal infections.

#### 2.2.3. Telavancin

Telavancin (trade name Vibativ), developed by Theravance Biopharma Antibiotics, Inc., is a lipoglycopeptide antibiotic with rapid bactericidal activity against both aerobic and anaerobic Gram-positive bacteria, including MRSA, vancomycin-intermediate *S. aureus* (VISA) and penicillin-resistant *S. pneumoniae* [89,90,91]. It is a semi-synthetic derivative of vancomycin whose dual mechanism of action involves inhibiting cell wall synthesis of peptidoglycan chain and disrupting membrane barrier function by dissipating its potential [92].

Telavancin has a high plasma protein binding (average 90%; mainly with albumin) and an average half-life of 8 h [15]. Its main route of elimination is renal excretion and dose adjustment is needed in cases with renal impairment [15] Telavancin has good penetration in the ELF and alveolar macrophages of healthy subjects, resulting in concentrations 8 to 85-fold above MIC_90_ of MRSA strains [91]. In vitro, telavancin is not deactivated by the presence of the alveolar surfactant but remains active [91].

Two identical, randomized phase III trials were conducted to compare telavancin (10 mg/kg IV every 24 h) and vancomycin (1 g IV every 12 h) for the treatment of cSSSI due to MRSA (NCT00091819 (ATLAS1), NCT00107978 (ATLAS2)) [15,93]. In patients with MRSA infections, cure rates were 91% when treated with telavancin and 86% when treated with vancomycin [94]. Cure rates for telavancin and vancomycin treatment arms were comparable (88.3% vs. 87.1%) [94]. Adverse events that resulted in discontinuation of therapy occurred in 8% and 6% when treated with telavancin and vancomycin respectively [94]. When given once daily, telavancin is at least as effective as vancomycin for cSSSI, including infections caused by MRSA [94]. Adverse events were similar between groups in terms of type and severity, however mild taste disturbance, nausea and vomiting and elevated serum creatinine concentration was apparent in the telavancin group, whilst pruritus occurred in the vancomycin treatment [94].

Another two identical, randomized, double-blind phase III non-inferiority studies (NCT00107952 [ATTAIN1], NCT00124020 [ATTAIN2])) compared the safety and effectiveness of telavancin with vancomycin for the treatment of HAP in adult patients [89]. Telavancin resulted in higher cure rates for *S. aureus* infections than vancomycin, and comparable cure rates in MRSA infections [89]. However, vancomycin achieved higher cure rates in patients with mixed Gram positive and Gram-negative infections [89]. Mortality rates for telavancin and vancomycin-treated patients were dissimilar in the two studies, but there was no statistical significance in either of them: 21.5% vs. 16.6% (95% CI for the difference, −0.7% to 10.6%) for the NCT00107952 and 18.5% vs. 20.6% (95% CI for the difference, −7.8% to 3.5%) for the NCT00124020 [89]. Telavancin resulted in increased serum creatinine levels more frequently (16% vs. 10%) compared to the vancomycin group, nevertheless, incidence and types of other adverse events, such as anemia, abnormal serum potassium levels and hepatic enzyme abnormalities, were comparable between the two groups of ATTAIN studies [89]. Baseline renal status should be considered before treatment initiation, while renal function monitoring is necessary for all patients on telavancin [89]. Moreover, it should be noted that the effectiveness of telavancin appears to be lower in patients with pre-existing moderate renal impairment (creatinine clearance <50 mL/min) [95].

The FDA approved telavancin for its use in cSSSI in September 2009 and in June 2013, approved its use in HAP and VAP caused by *S. aureus.* Telavancin has been withdrawn from use in the European Union due to insufficient data to conclude a positive benefit-risk balance for use in cSSSI in adults [96]. Phase IV studies involving the pharmacokinetics of telavancin use in cystic fibrosis (NCT03172793) and pediatric patients (NCT02013141) are currently recruiting and are estimated to be completed in mid-2019 and December 2020, respectively.

The rapid bactericidal activity, including VISA strains, along with its favorable pharmacokinetic characteristics (high protein-binding, long half-life, post-antibiotic effect), makes telavancin a promising antibacterial agent that complements the armamentarium against HAP and VAP from Gram-positive pathogens, especially MRSA. More studies are warranted to evaluate further indications of telavancin and its future role in the clinical setting.

### 2.3. Oxazolidinones

#### Tedizolid Phosphate

Tedizolid Phosphate (formerly torezolid, trade name Sivextro), developed by Cubist Pharmaceuticals, is a novel phosphate ester prodrug of tedizolid, an oxazolidinone-class antibiotic [97]. Tedizolid, exerts bacteriostatic activity by binding to the 23S ribosomal RNA (rRNA) of the 50S subunit of the bacterial ribosome and thus inhibiting protein synthesis [24]. Compared to linezolid which has a similar mechanism of action, it binds stronger to the active site due to its unique D-ring substituent providing additional hydrogen bonds [24].

In vitro studies have found that tedizolid has high antibacterial activity against Gram-positive microorganisms resistant to commonly used antibiotics, such as linezolid and methicillin [98]. The unique D-ring and a hydroxymethyl group has important contribution to tedizolid’s activity against linezolid-resistant strains [24]. It should be noted, however, that although tedizolid is active against linezolid-resistant pathogens with plasmid-borne and transposon associated chloramphenicol-florfenicol resistance (*cfr*) gene, it has cross-resistance to linezolid when mutations in chromosomal genes encoding 23S rRNA or ribosomal proteins (L3 and L4) are present [99,100]. One study showed that the antibacterial activity of tedizolid was four to eight times higher than linezolid with 302 MRSA strains and 220 vancomycin-intermediate *Enterococcus* spp. Strains [101]. Overall, tedizolid maintained a potent level of activity against target pathogens, especially staphylococci [101]. This increased potency allows for once daily dosing with reduced total dosages. Only 13 of 6884 strains had a tedizolid MIC values ≥1 μg/mL, whilst 99.8% of isolates were inhibited at MIC values ≤0.5 μg/mL [89,102]. Similarly, in neutropenic mouse thigh models of MSSA and MRSA infections, tedizolid demonstrated potent activity against one MSSA strain and two MRSA strains, outperforming linezolid in each case [90,103]. The study concluded that tedizolid demonstrated superiority in microbiological effect relative to linezolid even after MIC, protein binding and pharmacokinetics were adjusted for [103].

The phosphate group of tedizolid’s prodrug increases its bioavailability by increasing its water solubility [24]. Thus, following oral or intravenous administration, tedizolid phosphate is rapidly converted by endogenous phosphatases to tedizolid [24]. Tedizolid accumulates in macrophages of the peripheral blood and of the alveoli [24]. It also has extensive penetration into both extracellular and intracellular pulmonary compartments resulting in higher concentrations (>20-fold) in the ELF and alveolar macrophages compared to free plasma concentrations [24,104]. Studies performed in obese patients, patients with any degree of renal failure or liver failure and those over 65 years of age, demonstrated similar pharmacokinetics and no difference in adverse events, therefore no dose adjustment is required, even though approximately 35% is excreted by the kidneys [105].

Two pivotal phase III trials were conducted to compare the safety profiles of tedizolid phosphate to linezolid in the treatment of ABSSSI [106]. ESTABLISH-1, a randomized, double-blind, multi-center phase III trial was conducted in 667 adults ≥18 years of age with ABSSSI (NCT01170221) [106]. Tedizolid phosphate was found to be statistically non-inferior to linezolid in the early stages of clinical response (48 to 72 h) after therapy commencement [106]. The overall incidence of serious adverse events was similar after a 10-day course of either tedizolid phosphate and linezolid [106]. ESTABLISH-2, a randomized, double-blind, non-inferiority phase III trial investigating the use of tedizolid for six days versus linezolid for 10 days was conducted in patients ≥12 years of age with ABSSSI [107]. Similar to results from ESTABLISH-1, tedizolid achieved early clinical response and reached the prespecified non-inferiority margin [107]. Moreover, the tedizolid group experienced less frequent gastrointestinal adverse events than the linezolid group, and one patient in the tedizolid group experienced a treatment-emergent adverse event versus four patients in the linezolid group [107]. The most common adverse reactions (>2%) occurring in patients treated with tedizolid were nausea (8%), headache (6%), diarrhea (4%), vomiting (3%), and dizziness (2%) [18]. Tedizolid may have lower hematologic toxicity compared with linezolid. In the ESTABLISH 1 & 2 trials, thrombocytopenia was observed in 2.3% of the tedizolid treated patients, compared with 4.9% of the linezolid treated patients [106]. However, this finding may reflect the shorter tedizolid dosing regimen used in these trials [106]. As the number of patients treated with tedizolid increases, the assessment of thrombocytopenia should be investigated with real-world safety studies [108].

Tedizolid phosphate was approved by the FDA in June 2014 for the treatment of adult patients with ABSSSI [109,110]. It was also approved by the EMA in March 2015 for the same indication [110]. A phase III non-inferiority trial (NCT02019420) that compared tedizolid with linezolid for the treatment of Gram-positive ventilated nosocomial pneumonia was completed in June 2018 and results are pending [111]. The two study groups received either tedizolid 200 mg IV once daily for 7 days (or 14 for concurrent bacteremia) or linezolid 600 mg IV twice daily for 10 days (or 14 days for concurrent bacteremia and the primary outcome was 28-day all-cause mortality [111]. Currently, a phase I clinical trial (NCT03217565) determining the single-dose pharmacokinetics of IV and oral tedizolid phosphate in pediatric participants with Gram-positive infections started in February 2019 and is expected to be completed in July 2021 [20]. Another randomized phase III trial (NCT03176134) evaluating the safety, tolerability and efficacy of tedizolid phosphate versus a comparator agent in participants <12 years of age with ABSSSI started in January 2019 and is expected to be completed in September 2021 [20].

Tedizolid is the first oxazolidinone administered only once-daily. The convenient dosing scheme, the lack of need for dose adjustment when switching from IV to oral administration, along with its action against linezolid-resistant strains and the lower frequency of thrombocytopenia, makes tedizolid a promising antibacterial agent against severe infections from Gram-positive pathogens. However, further assessment is needed of thrombocytopenia development in the clinical setting.

### 2.4. Quinolones

#### 2.4.1. Besifloxacin

Besifloxacin (trade name Besivance), developed by SSP Co. Ltd. and sold to Bausch & Lomb in 2003, is a fluoroquinolone antibiotic that was approved by the FDA in May 2009 for bacterial conjunctivitis [112]. Similar to other fluoroquinolones, besifloxacin’s mechanism of action involves inhibition of DNA topoisomerases including DNA gyrase and topoisomerase IV, thereby inhibiting DNA synthesis [113]. Besifloxacin exhibits greater in vitro activity against both enzymes in *S. pneumoniae* compared to moxifloxacin and ciprofloxacin [112]. Besifloxacin has a wide spectrum of antibacterial activity that covers Gram-positive, Gram-negative and anaerobic organisms [112,114,115]. It demonstrates particularly more rapid in vitro bactericidal effects on the common ocular pathogens *S. aureus*, *S. epidermidis*, *S. pneumoniae*, and *Haemophilus influenzae* compared to moxifloxacin and gatifloxacin [112,114,115]. The Antibiotic Resistance Monitoring in Ocular microorganisms (ARMOR) study in the United States found that besifloxacin exhibited the lowest MIC_90_ against staphylococcal (including methicillin-resistant isolates) and streptococcal isolates among fluoroquinolones, with a MIC_90_ comparable to vancomycin for MRSA [116]. Multiple studies showed that the emergence of besifloxacin-resistant *S. aureus* and *S. pneumoniae* occurred at a much lower rate than other fluoroquinolones due to its non-preferential binding for both DNA gyrase and topoisomerase IV, resulting in a reduced risk for spontaneous resistance [117,118,119].

Besifloxacin is available as a 0.6% (6 mg/mL) ophthalmic suspension and is approved for topical ophthalmic use for bacterial conjunctivitis in patients with an age of one year and older [120]. The commonest adverse effects include blurred vision, eye pain, eye irritation, conjunctival redness, and eye pruritus (1.1–2.1% incidence) [121]. In regard to the safety and efficacy of besifloxacin in neonates, a phase III, small, multi-center, randomized, double-masked, parallel clinical trial (n = 33) found that both besifloxacin and gatifloxacin were safe and effective in treating neonatal bacterial conjunctivitis, with besifloxacin demonstrating earlier bacterial eradication with no adverse events reported [122]. The commonest adverse event associated with besifloxacin for topical ophthalmic use is conjunctival redness (2%) [19].

#### 2.4.2. Delafloxacin

Delafloxacin (trade name Baxdela), developed by Melinta Therapeutics Inc., is a novel fluoroquinolone which inhibits DNA gyrase and topoisomerase IV [20,123]. Its distinct chemical structure confers to delafloxacin a weakly acidic character that results in increased cellular penetration as well as increased bactericidal activity in the acidic environment of the infection site [124]. This enhanced antibacterial activity is contrary to the characteristics of the other members of the class that demonstrate decreased activity in acidic environments [124]. Moreover, compared to the other fluoroquinolones, delafloxacin exerts a more balanced inhibition of both DNA gyrase and topoisomerase IV that should theoretically decrease resistance selection [124].

Delafloxacin covers a broad-spectrum of Gram-positive pathogens, including *S. aureus* (including MRSA and MSSA isolates), *S. pneumoniae*, and most fluoroquinolone-resistant strains except enterococci [20,123]. Delafloxacin also has good antimicrobial activity against *Enterobacteriaceae*, but its activity against *P. aeruginosa* is weaker compared to that of ciprofloxacin. In vitro studies have shown that delafloxacin is highly potent against fluoroquinolone-susceptible *S. aureus* with a MIC_50_ of 0.004 mg/l compared to that of levofloxacin (0.25 mg/L) and moxifloxacin (0.06 mg/L), and fluoroquinolone-resistant *S. aureus* with a MIC_50_ of 0.25 mg/l compared to that of levofloxacin (16 mg/L) and moxifloxacin (4 mg/L) [125]. Another in vitro study found that the MIC_50_ of delafloxacin was 4- to 64-fold lower than that of ciprofloxacin, levofloxacin and moxifloxacin against 30 clinical MRSA strains [126]. In murine neutropenic lung infection models, delafloxacin showed significantly lower MIC against MRSA, MSSA and *S. pneumoniae*, when compared with azithromycin and levofloxacin [127].

Delafloxacin has high plasma protein binding (average 83%, mainly albumin) [124]. It is eliminated via the kidneys, therefore adjustment for intravenous dosing is required in patients with renal impairment but not hepatic impairment [128]. The bioavailability of delafloxacin oral formulation is 58.8%, with a volume of distribution of 30–48 L, a mean half-life of 3.7 h and liver glucuronidation as its primary metabolic pathway [20]. Delafloxacin, similarly to the other members of fluoroquinolone class, has high lung penetration with 13:1 ELF to free plasma concentration [124].

In one multi-center, randomized, double-blind phase II study (NCT00719810), delafloxacin was as effective as tigecycline for cSSSIs and was well tolerated [129]. In another randomized, double-blind phase II study (NCT01283581), delafloxacin demonstrated significantly higher clinical cure rates than vancomycin (mean difference −16.3%, 95% CI: −30.3% to −2.3%) against ABSSSIs caused by all clinical isolates except MRSA, in which cure rates for delafloxacin were comparable to vancomycin or linezolid [130]. In a multi-center, randomized, double-blind phase III study (NCT01984684), IV/oral monotherapy delafloxacin was non-inferior to IV vancomycin/aztreonam in patients with ABSSSIs, including those with MRSA infections, with the percentage of patients achieving ≥20% reduction in erythema area of the lesion being 83.7% versus 80.6% (95% CI −2.0 to 8.3%) at 48 to 72 h [131].

Its common side effects (≥2% incidence), similar to other fluoroquinolones, include tendinitis, tendon rupture, arthralgia, myalgia, peripheral neuropathy, and central nervous system effects including hallucinations, anxiety, depression, insomnia, severe headaches, and confusion [20]. Other more serious adverse reactions that led to study discontinuation include urticaria (0.3%) and hypersensitivity (0.3%) [20]. Although the available clinical evidence is rather limited, delafloxacin appears to be better tolerated compared with other commonly used fluoroquinolones [132].

Delafloxacin was approved by the FDA for the treatment of ABSSSIs in adults in June 2017 [133]. Melinta Therapeutics also submitted a Marketing Authorization Application (MAA) to the EMA for the same indication in March 2018 [134]. A multi-center, randomized, double-blind phase III study (DEFINE-CABP, NCT02679573) comparing delafloxacin to moxifloxacin for treating adults with CABP was completed in 2018 with results pending, while Melinta Therapeutics had submitted an NDA to the FDA for the indication for CABP in the same year [135,136]. Delafloxacin has received QIDP from FDA for cUTI [136].

The enhanced intracellular penetration and bactericidal activity in acidic environments, along with the dual mechanism of action that limits resistance development, makes delafloxacin a promising treatment for ABSSSIs, and in the future, depending on trials results, potentially for CABP.

#### 2.4.3. Ozenoxacin

Ozenoxacin (trade name Ozaenex/Xepi), developed by Ferrer International S.A., is a novel, non-fluorinated, topical quinolone that is bactericidal against many Gram-positive organisms, including MRSA, MSSA, MRSE and *S. pyogenes* [137]. In vitro, MIC_90_ values against bacteria such as MSSA, MRSA, *S. epidermis* was between two- and >16,000-fold greater than compared to other commonly prescribed antibiotics, such as ofloxacin, levofloxacin, erythromycin and gentamicin [138]. Previous trials demonstrated a complete lack of systemic absorption and no formation of metabolites when ozenoxacin was applied topically, likely contributing to the absence of drug interactions [137]. As a result, the elimination of ozenoxacin has not been investigated in humans [139]. Previous studies conducted to determine the toxicity of orally administered ozenoxacin have demonstrated non-toxicity in the brain, thymus, liver, lungs and kidney after adequate systemic exposure in juvenile dogs [140]. Furthermore, no dose adjustment is required for geriatrics or people with hepatic and renal impairment [139].

The FDA approved ozenoxacin in November 2017 for the treatment of impetigo caused by *S. aureus* and *S. pyogenes* in both adults and pediatric patients older than two months of age [21,137]. This was based on two multi-center, randomized, phase III clinical trials evaluating the safety and efficacy of ozenoxacin cream in patients with non-bullous or bullous impetigo (NCT01397461 and NCT02090764) [137]. Ozenoxacin demonstrated superior clinical success compared to the placebo after five days of therapy and superior microbiological success after two days of therapy [141]. Ozenoxacin was well tolerated with only one of 206 patients experiencing rosacea and seborrheic dermatitis which was not considered a serious adverse event [141].

### 2.5. Tetracyclines

#### Omadacycline

Omadacycline (trade name Nuzyra), developed by Paratek Pharmaceuticals, is a tetracycline antibiotic belonging to the aminomethylcycline subclass, with improved oral bioavailability compared to tigecycline, and its antibacterial profile and efficacy are superior to the earlier members of the class, such as tetracycline, doxycycline, and minocycline [142]. Omadacycline binds to the 30S ribosomal subunit and inhibits protein synthesis and is active against certain antibiotic resistance mechanisms such as tetracycline efflux and ribosome protection [143]. In vitro, omadacycline is active against bacteria associated with ABSSSIs and CABP including MRSA, penicillin-resistant and multidrug-resistant *S. pneumoniae*, and vancomycin-resistant *Enterococcus* spp. [144]. It also has a broad range of activity against Gram-negative bacteria, with the main exclusion of *P. aeruginosa*.

Omadacycline has low plasma protein binding and a half-life of approximately 16–17 h, which supports once daily dosing scheme [25]. It is eliminated via the kidneys but does not require dose adjustment in either kidney or liver impairments [22]. Omadacycline has demonstrated good lung penetration with the ratio of ELF and alveolar cells to total plasma concentrations being 1.47 and 25.8 respectively, compared with those of tigecycline (1.71 and 20.8, respectively) [145].

A phase II study indicated that omadacycline was well-tolerated and effective against cSSSIs compared with linezolid [146]. A phase III study (NCT02378480) demonstrated that omadacycline was effective and non-inferior to linezolid for the treatment of ABSSSIs (rate of early clinical response 84.8% vs. 85.5%, respectively), and was generally safe and well-tolerated following both IV and oral dosing, with a similar safety profile to linezolid (adverse events 48.3% in the omadacycline group vs. 45.7% in the linezolid group, respectively; with gastrointestinal being the most frequent in both groups) [147,148,149]. Rates of clinical response at the post-treatment evaluation were similar in the omadacycline and linezolid groups among patients with monomicrobial gram-positive infections (87.8% and 84.8%, respectively), polymicrobial gram-positive infections (74.2% and 81.5%), and polymicrobial mixed infections (80.5% and 75.9%). Clinical response at the post-treatment evaluation in the small subgroup of patients with bacteremia occurred in 82% of patients (9 of 11) who received omadacycline and 100% of patients (9 of 9) who received linezolid. In both groups, the efficacy of omadacycline and linezolid against MRSA (83 vs. 86%, respectively) and MSSA (84 vs. 82%, respectively) infections were similar [149]. Another phase III study (NCT02531438) found that oral omadacycline was safe, well-tolerated and non-inferior to moxifloxacin in treating CABP with a clinical success rate of 92.9% versus 90.4% (95% CI: −1.7 to 6.8) [150]. Omadacycline demonstrated non-inferiority to moxifloxacin for early clinical response (83.1% vs. 82.7%, respectively) [151]. The analysis of the microbiologic ITT population also resulted in similar clinical response rates (89.2% and 87.4%), while similar efficacy in the two treatment groups was observed within subgroups based on Pneumonia Severity Index (PSI) risk class [151]. With regards to adverse events after treatment initiation, omadacycline had 41.15% compared to 48.5% of moxifloxacin group, with gastrointestinal events being the most frequent (10.2% vs. 18.0%, respectively (the largest difference was in the incidence of diarrhea, i.e., 1.0% vs. 8.0%, respectively) [151]. Although eight deaths occurred in the omadacycline group and four in the moxifloxacin group, these all occurred in patients older than 65 years of age and were due to progression of the underlying pneumonia or respiratory compromise, cardiac or vascular events and cancer [151].

In October 2018, the FDA approved omadacycline for the indications of CABP and ABSSSIs in both IV and tablet formulations [22]. The submission of the MAA from Paratek Pharmaceuticals was also accepted in the same month by the EMA [152]. A phase II study (NCT03425396) that compared per os administration of omadacycline versus nitrofurantoin for cystitis and uncomplicated UTI, was very recently completed and topline data are expected to be announced in the second half of 2019 [152]. Another phase II clinical trial (NCT03757234) testing omadacycline (IV or IV/PO) efficacy versus levofloxacin (IV/PO) in treating acute pyelonephritis is expected to be completed in September 2019 [153].

With a very wide anti-bacterial spectrum, minimal drug-drug interaction, a favorable safety profile and the availability of both IV and oral formulations, omadacycline seems to be a promising alternative for the treatment of CABP and ABSSSI, while further studies will evaluate its clinical role for further indications.

## 3. Conclusions

Increasing multidrug-resistance to Gram-positive pathogens, particularly those caused by MRSA, VRE and *S. pneumoniae*, has become a major problem in hospital environments, resulting in significant morbidity and mortality [154]. As antibiotics are rendered resistant to bacteria, this leads to the overuse of empiric therapy as well as the need to resort to potentially more toxic agents [154].

The antimicrobial agents discussed above are highly significant in the treatment of MDR infections, however, as for any emerging antibiotic agent, resistance is likely to develop. Only through appropriate dosing, utilization and careful monitoring for the emergence of antimicrobial resistance can these current antimicrobial drugs continue to treat Gram-positive pathogens in the future [155]. Future trials should be conducted to investigate these drugs for further indications of use and unanticipated adverse effects.

## Figures and Tables

**Table 1:** Summary of novel antibiotics against Gram-positive bacteria approved by FDA and/or EMA during the last decade.

Drug	Approval Time	Antibiotic Class	Company	Spectrum Against Organisms	Indication	Dose ^1^	Comments/Warnings ^2^
Ceftaroline (Teflaro/Zinforo)	FDA: October 2010 EMA: August 2012	Cephalosporin	Allergan Pharmaceutical Industries Ltd. (US/Canada); Takeda Pharmaceutical Company Ltd. (Japan); Pfizer (globally except US/Canada/Japan)	ABSSSI: MRSA, MSSA, *S. pyogenes*, *S. agalactiae* CABP: MSSA, *S. pneumoniae*, *H. influenzae*	FDA: CABP and ABSSSI EMA: CABP and cSSSI	^3^ IV: 600 mg over 5 to 60 min every 12 h [13]	Serious anaphylactic reactions have been reported with beta-lactam antibiotics Direct Coombs’ test seroconversion has been reported; if anaemia develops during or after therapy, consider drug-induced haemolytic anaemia and ceftaroline
Ceftobiprole (Zevtera/Mabelio)	EMA: October 2013	Cephalosporin	Basilea Pharmaceutica Ltd.	MRSA, ampicillin-susceptible enterococci and penicillin-resistant pneumococci	EMA: HAP (excluding VAP) and CABP	IV: 500 mg over 2 h every 8 h [14]	Serious anaphylactic reactions have been reported in patients receiving beta-lactam antibiotics
Telavancin (Vibativ)	FDA: September 2009	Lipoglycopeptide	Theravance Biopharma Antibiotics, Inc.,	MRSA, vancomycin-intermediate *S. aureus* and penicillin-resistant *S. pneumoniae*	FDA: cSSSI, HAP (including VAP)	IV: 10 mg/kg over 60 min every 24 h for 7–14 days (cSSSI) and 7–21 days (HAP/VAP) [15]	Decreased efficacy with moderate/severe pre-existing renal impairment Interferes with some coagulation tests e.g., prothrombin time, international normalised ratio QTc prolongation Serious and potentially fatal hypersensitivity reactions Infusion-related reactions
Dalbavancin (Dalvance/ Xydalba)	FDA: May 2014	Lipoglycopeptide	Durata Therapeutics (acquired by Actavis in 2014)	MRSA, *S. pyogenes*, *S. agalactiae* and *E. faecalis* strains susceptible to vancomycin	FDA: ABSSSI	IV: 1000 mg over 30 min followed one week later by 500 mg over 30 min [16]	Serious anaphylactic and skin reactions have been reported with glycopeptides Rapid IV glycopeptide infusion can cause upper body flushing, urticaria, pruritis and/or rash ALT elevations have been reported
Oritavancin (Orbactiv)	FDA: August 2014 EMA: March 2015	Glycopeptide	Melinta Therapeutics Inc.	MSSA, MRSA, VRE and vancomycin-intermediate and vancomycin-resistant staphylococci	FDA: ABSSSI EMA: ABSSSI	IV: 1200 mg single dose over 3 h [17]	Co-administration with warfarin may increase warfarin exposure and increase bleeding risk Oritavancin administration may artificially prolong aPTT for up to 48 h and prolong PT/INR for up to 24 h Hypersensitivity and infusion related reactions including pruritus, urticaria and flushing have been reported ^4^ A higher incidence of osteomyelitis reported in the oritavancin treated ABSSSI arm than vancomycin-treated arm
Tedizolid Phosphate (Sivextro)	FDA: June 2014 EMA: March 2015	Oxazolidinone	Cubist Pharmaceuticals	MRSA, vancomycin-intermediate *Enterococcus* spp.	FDA: ABSSSI EMA: ABSSSI	IV: 200 mg single dose over 1 h for 6 days ^5^ PO: 200 mg once daily [18]	Safety and efficacy not adequately evaluated in neutropenic patients
Besifloxacin (Besivance)	FDA: June 2009	Fluoroquinolone	SSP Co. Ltd.	MRSA, *S. epidermidis*, *S. pneumoniae*, and *H. influenzae*	FDA: bacterial conjunctivitis	Instill one drop in the affected eye(s) 3 times a day, four to 12 h apart for 7 days [19]	Not for injection into the eye Prolonged use may result in the overgrowth of non-susceptible organisms resulting in a super-infection Avoid contact lens wear during course of therapy
Delafloxacin (Baxdela)	FDA: June 2017	Fluoroquinolone	Melinta Therapeutics Inc.	*S. aureus* (including MRSA), *S. pneumoniae*, other fluoroquinolone resistant strains (Ineffective against Fluoroquinolone-resistant enterococci)	FDA: ABSSSI	IV: 300 mg over 1 h every 12 h PO: 450 mg tablet every 12 h for 5 to 14 days [20]	Hypersensitivity reactions may occur after first or subsequent doses
Ozenoxacin (Ozaenex/Xepi)	FDA: December 2017	Non-fluorinated quinolone	Ferrer Internacional S.A.	MRSA, MSSA, MRSE and *S. pyogenes*	FDA: impetigo	Topical: apply a thin layer to the affected area twice daily for 5 days [21	Prolonged use of ozenoxacin may result in the overgrowth of non-susceptible organisms resulting in a super-infection
Omadacycline (Nuzyra)	FDA: October 2018	Tetracycline	Paratek Pharmaceuticals	MRSA, penicillin-resistant and multidrug-resistant *S. pneumoniae*, and vancomycin-resistant *Enterococcus* spp.	FDA: CABP, ABSSSI	Duration: 7–14 days Loading IV Day1: 200 mg over 1 h once daily or 100 mg over 30 min twice daily *Maintainance:* 100 mg over 30 min or 300 mg po once daily ^6^ *Loading PO (ABSSSI)* Day 1&2: 450 mg once daily *Maintainance PO (ABSSSI)* 300 mg once daily [22]	Commonest adverse reactions: Nausea, vomiting, hypertension, headache, diarrhea, insomnia and constipation Mortality imbalance observed in the CABP clinical trial: 8 deaths in the omadacycline group vs. 4 in the moxifloxacin group Omadacycline use during tooth development (last half of pregnancy, infancy and childhood >8 years) may cause permanent teeth discoloration and enamel hypoplasia Omadacycline use during the 2nd and 3rd trimester of pregnancy, infancy and childhood >8 years may cause reversible bone growth inhibition

Notes: ^1^ All dosing regimens are indicated in adult patients >18 years old with no renal or hepatic impairment. See individual drug label for dosing regimens in other populations. ^2^ *C. difficile*-associated diarrhea has been reported with nearly all systemic antibacterial agents; ^3^ 600 mg every 8 h should be considered as it is safe and is expected to be a better dosing scheme for critically ill patients with normal or augmented renal clearance [23]. ^4^ Patients with ABSSSIs treated with oritavancin should be monitored closely for symptoms and signs of osteomyelitis and in case of osteomyelitis diagnosis, appropriate treatment should be initiated promptly ^5^ Tedizolid has excellent oral bioavailability (more than 90%) and thus there is no need for dosage adjustment when switching from intravenous to oral administration [24]. ^6^ Omadacycline per os should be administered after at a fasted state and certain foods (e.g., dairy products, to be avoided for at least 4 h after dosing [25].

## Supplementary Materials

The following are available online at https://www.mdpi.com/2076-2607/7/8/270/s1, Figures S1–S10: Chemical structures.

## Abbreviations

ABSSSI (Acute Bacterial Skin and Skin Structure Infection), CABP (community-acquired bacterial pneumonia), cSSSI (complicated skin and skin structure infection), EMA (European Medicine Agency), FDA (Federal Drug Administration), HAP (hospital-acquired pneumonia), IV (intravenous), PO (per oral), VAP (ventilator-associated pneumonia).
