# Supplementary material for: Novel Antibiotics for Multidrug-Resistant Gram-Positive Microorganisms
Source: Microorganisms. 2019 Aug 18;7(8):270. doi: 10.3390/microorganisms7080270 (PMC6723731; doi:10.3390/microorganisms7080270)
Supplement: Supplementary file 1 [file microorganisms-07-00270-s001.pdf]

Supplement

Reference: National Center for Biotechnology Information. The PubChem Project. National Center for Biotechnology Information; 2019. [Available from: https://pubchem.ncbi.nlm.nih.gov/] [accessed 01/08/2019]

1. Ceftaroline

Chemical name: Ceftaroline
PubChem CID: 56841980
Molecular Formula: $C_{24}H_{25}N_8O_{10}PS_4$
Molecular Weight: 744.7 g/mol
2D Structure:

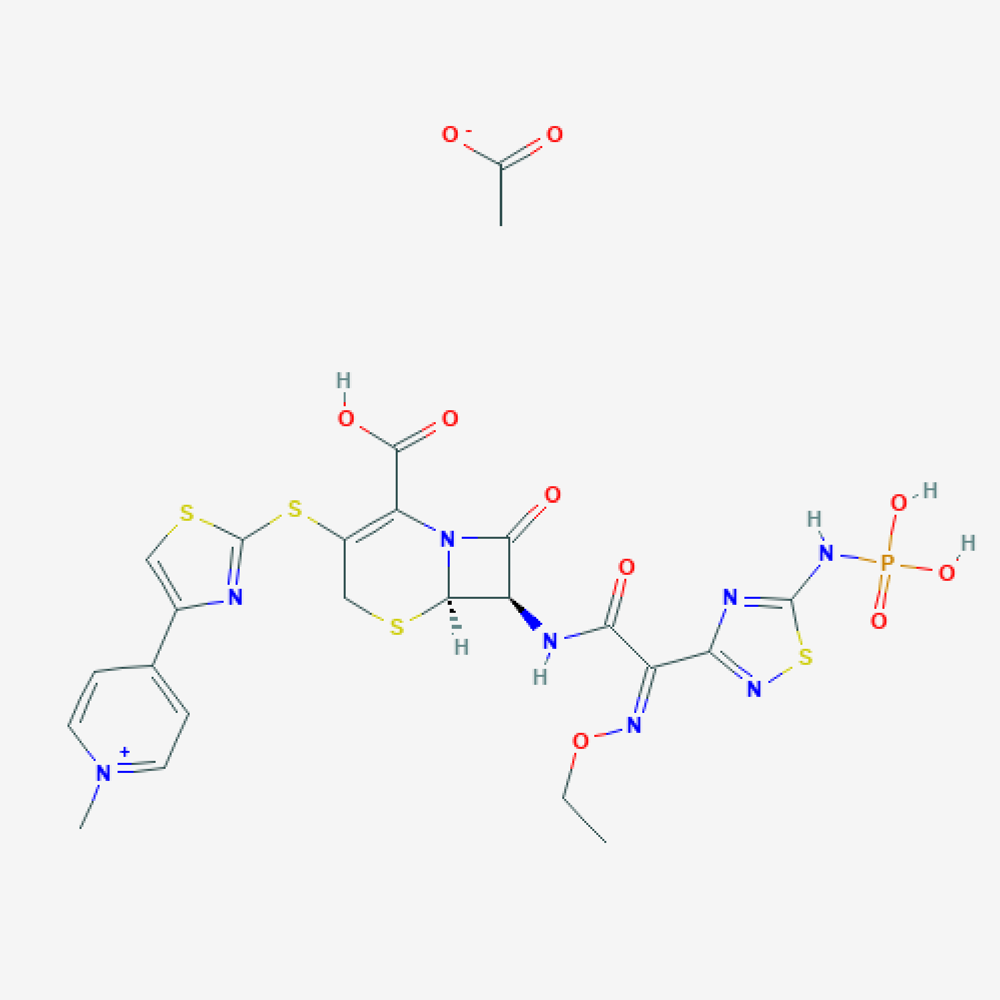

IUPAC Name:
(6*R*,7*R*)-7-[[(2*Z*)-2-ethoxyimino-2-[5-(phosphonoamino)-1,2,4-thiadiazol-3-yl]acetyl]amino]-3-[[4-(1-methylpyridin-1-ium-4-yl)-1,3-thiazol-2-yl]sulfanyl]-8-oxo-5-thia-1-azabicyclo[4.2.0]oct-2-ene-2-carboxylic acid;acetate

2. Ceftobiprole

Chemical name: Ceftobiprole
PubChem CID: 135413542
Molecular Formula: $C_{20}H_{22}N_8O_6S_2$
Molecular Weight: 534.6 g/mol
2D Structure:

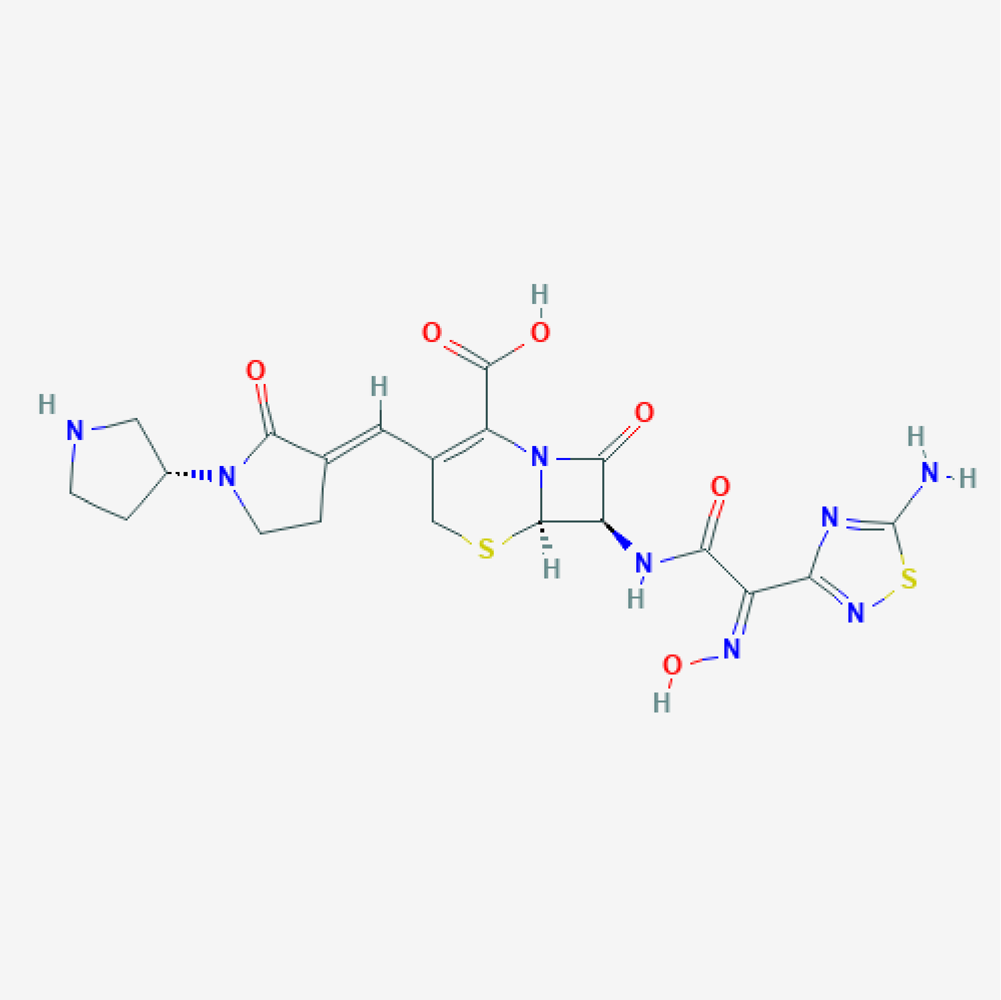

IUPAC Name:
(6*R*,7*R*)-7-[[(2*Z*)-2-(5-amino-1,2,4-thiadiazol-3-yl)-2-hydroxyiminoacetyl]amino]-8-oxo-3-[(*E*)-[2-oxo-1-[(3*R*)-pyrrolidin-3-yl]pyrrolidin-3-ylidene]methyl]-5-thia-1-azabicyclo[4.2.0]oct-2-ene-2-carboxylic acid

3. Dalbavancin

Chemical name: Dalbavancin
PubChem CID: 23724878
Molecular Formula: $C_{88}H_{100}Cl_2N_{10}O_{28}$
Molecular Weight: 1816.7 g/mol
2D Structure:

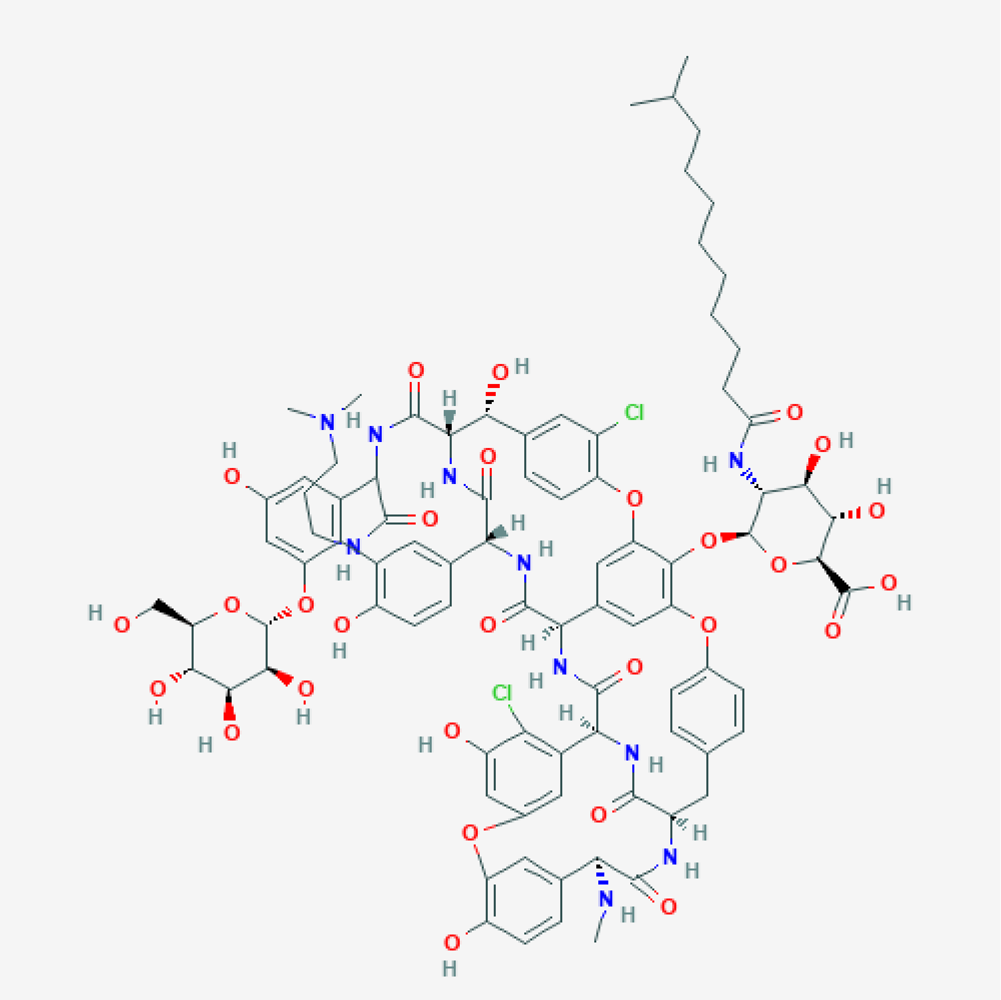

IUPAC Name:
(2*S*,3*S*,4*R*,5*R*,6*S*)-6-[[(1*S*,2*R*,19*R*,22*R*,34*S*,37*R*,40*R*)-5,32-dichloro-52-[3-(dimethylamino)propylcarbamoyl]-2,26,31,44,49-pentahydroxy-22-(methylamino)-21,35,38,54,56,59-hexaoxo-47-[(2*R*,3*S*,4*S*,5*S*,6*R*)-3,4,5-trihydroxy-6-(hydroxymethyl)oxan-2-yl]oxy-7,13,28-trioxa-20,36,39,53,55,58-hexazaundecacyclo[38.14.2.2^{3,6}.2^{14,17}.2^{19,34}.1^{8,12}.1^{23,27}.1^{29,33}.1^{41,45}.0^{10,37}.0^{46,51}]hexahexaconta-3,5,8,10,12(64),14(63),15,17(62),23(61),24,26,29(60),30,32,41(57),42,44,46(51),47,49,65-henicosaen-64-yl]oxy]-3,4-dihydroxy-5-(10-methylundecanoylamino)oxane-2-carboxylic acid

4. Oritavancin

Chemical name: Oritavancin

PubChem CID: 16136912

Molecular Formula: $C_{86}H_{97}Cl_3N_{10}O_{10}$

Molecular Weight: 1793.1 g/mol

2D Structure:

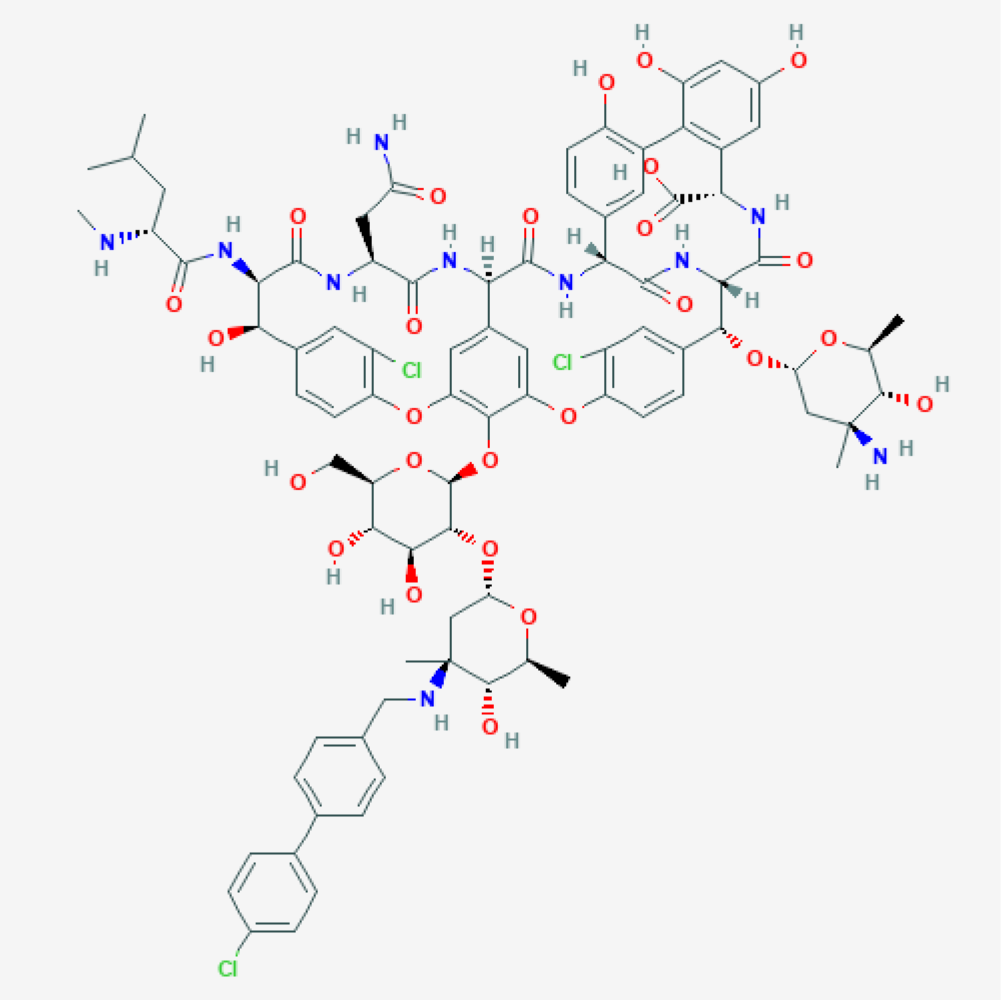

IUPAC Name:

(1*S*,2*R*,18*R*,19*R*,22*S*,25*R*,28*R*,40*S*)-2-[(2*R*,4*S*,5*R*,6*S*)-4-amino-5-hydroxy-4,6-dimethyloxan-2-yl]oxy-22-(2-amino-2-oxoethyl)-5,15-dichloro-48-[(2*S*,3*R*,4*S*,5*S*,6*R*)-3-[(2*S*,4*S*,5*R*,6*S*)-4-[[4-(4-chlorophenyl)phenyl]methylamino]-5-hydroxy-4,6-dimethyloxan-2-yl]oxy-4,5-dihydroxy-6-(hydroxymethyl)oxan-2-yl]oxy-18,32,35,37-tetrahydroxy-19-[[(2*R*)-4-methyl-2-(methylamino)pentanoyl]amino]-20,23,26,42,44-pentaoxo-7,13-dioxa-21,24,27,41,43-pentazaoctacyclo[26.14.2.$2^{3,6}$.$2^{14,17}$.$1^{8,12}$.$1^{29,33}$.$0^{10,25}$.$0^{34,39}$]pentaconta-3,5,8,10,12(48),14,16,29(45),30,32,34(39),35,37,46,49-pentadecaene-40-carboxylic acid

5. Telavancin

Chemical name: Telavancin
PubChem CID: 3081362
Molecular Formula: $C_{80}H_{106}Cl_2N_{11}O_{27}P$
Molecular Weight: 1755.6 g/mol
2D Structure:

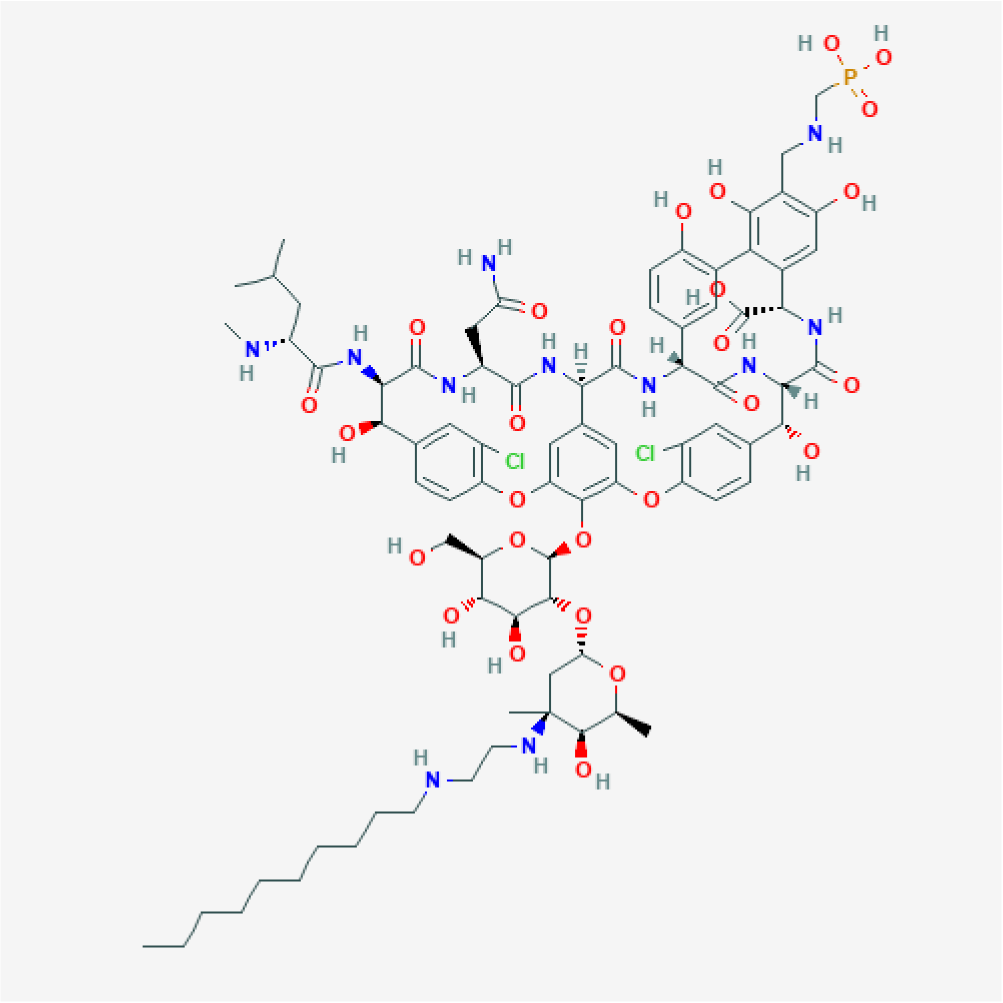

IUPAC Name:
(1*S*,2*R*,18*R*,19*R*,22*S*,25*R*,28*R*,40*S*)-22-(2-amino-2-oxoethyl)-5,15-dichloro-48-[(2*S*,3*R*,4*S*,5*S*,6*R*)-3-[(2*S*,4*S*,5*S*,6*S*)-4-[2-(decylamino)ethylamino]-5-hydroxy-4,6-dimethyloxan-2-yl]oxy-4,5-dihydroxy-6-(hydroxymethyl)oxan-2-yl]oxy-2,18,32,35,37-pentahydroxy-19-[[(2*R*)-4-methyl-2-(methylamino)pentanoyl]amino]-20,23,26,42,44-pentaoxo-36-[(phosphonomethylamino)methyl]-7,13-dioxa-21,24,27,41,43-pentazaoctacyclo[26.14.2.$2^{3,6}$.$2^{14,17}$.$1^{8,12}$.$1^{29,33}$.$0^{10,25}$.$0^{34,39}$]pentaconta-3,5,8(48),9,11,14,16,29(45),30,32,34,36,38,46,49-pentadecaene-40-carboxylic acid

6. Tedizolid Phosphate

Chemical name: Tedizolid Phosphate

PubChem CID: 11476460

Molecular Formula: $C_{17}H_{16}FN_6O_6P$

Molecular Weight: 450.3 g/mol

2D Structure:

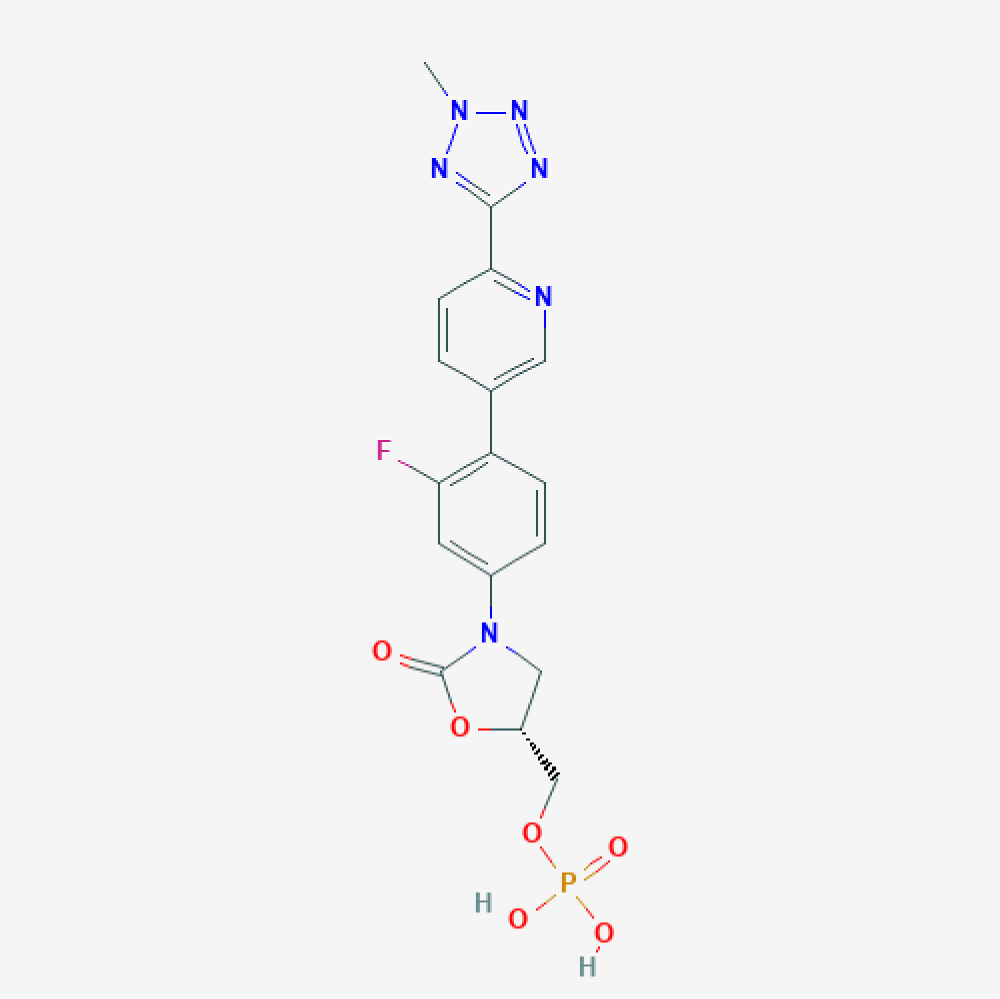

IUPAC Name:

[(5*R*)-3-[3-fluoro-4-[6-(2-methyltetrazol-5-yl)pyridin-3-yl]phenyl]-2-oxo-1,3-oxazolidin-5-yl]methyl dihydrogen phosphate

7. Besifloxacin

Chemical name: Besifloxacin
PubChem CID: 10178705
Molecular Formula: $C_{19}H_{21}ClFN_3O_3$
Molecular Weight: 393.8 g/mol
2D Structure:

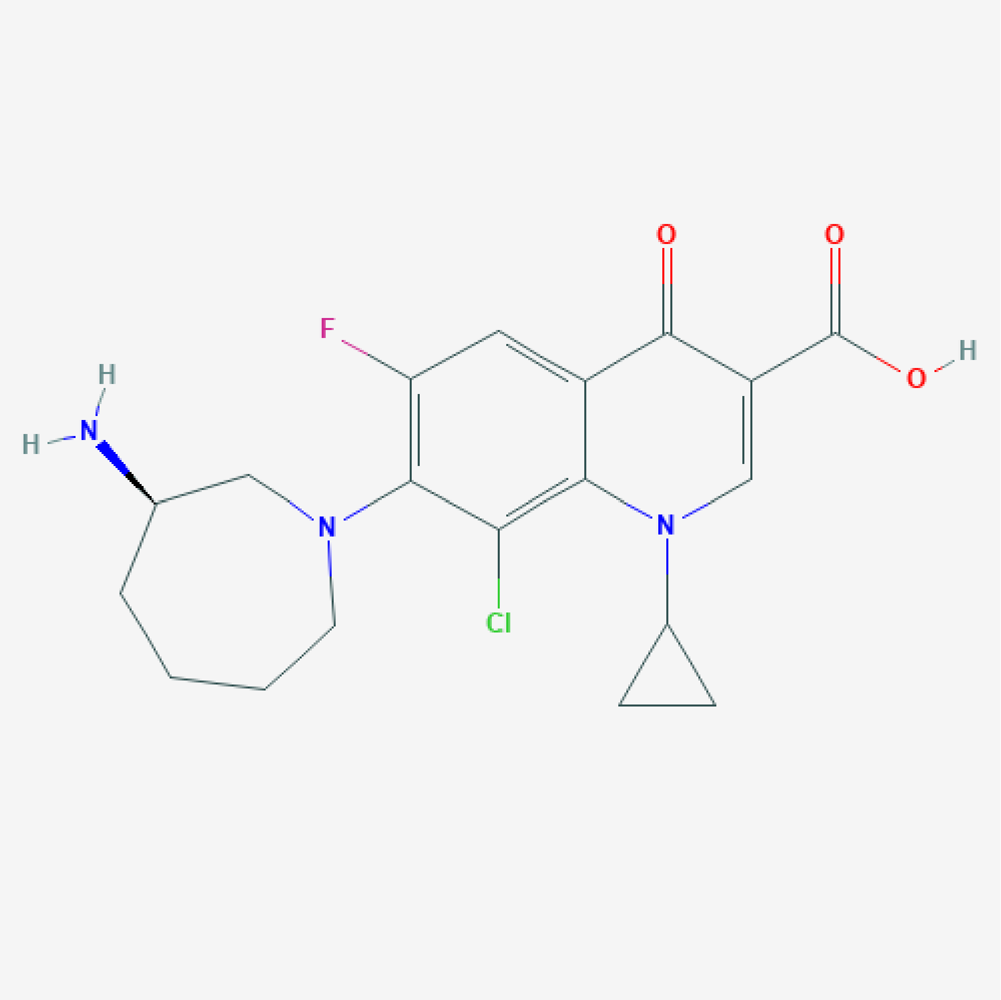

IUPAC Name:
7-[(3*R*)-3-aminoazepan-1-yl]-8-chloro-1-cyclopropyl-6-fluoro-4-oxoquinoline-3-carboxylic acid

8. Delafloxacin

Chemical name: Delafloxacin
PubChem CID: 487101
Molecular Formula: $C_{18}H_{12}ClF_3N_4O_4$
Molecular Weight: 440.8 g/mol
2D Structure:

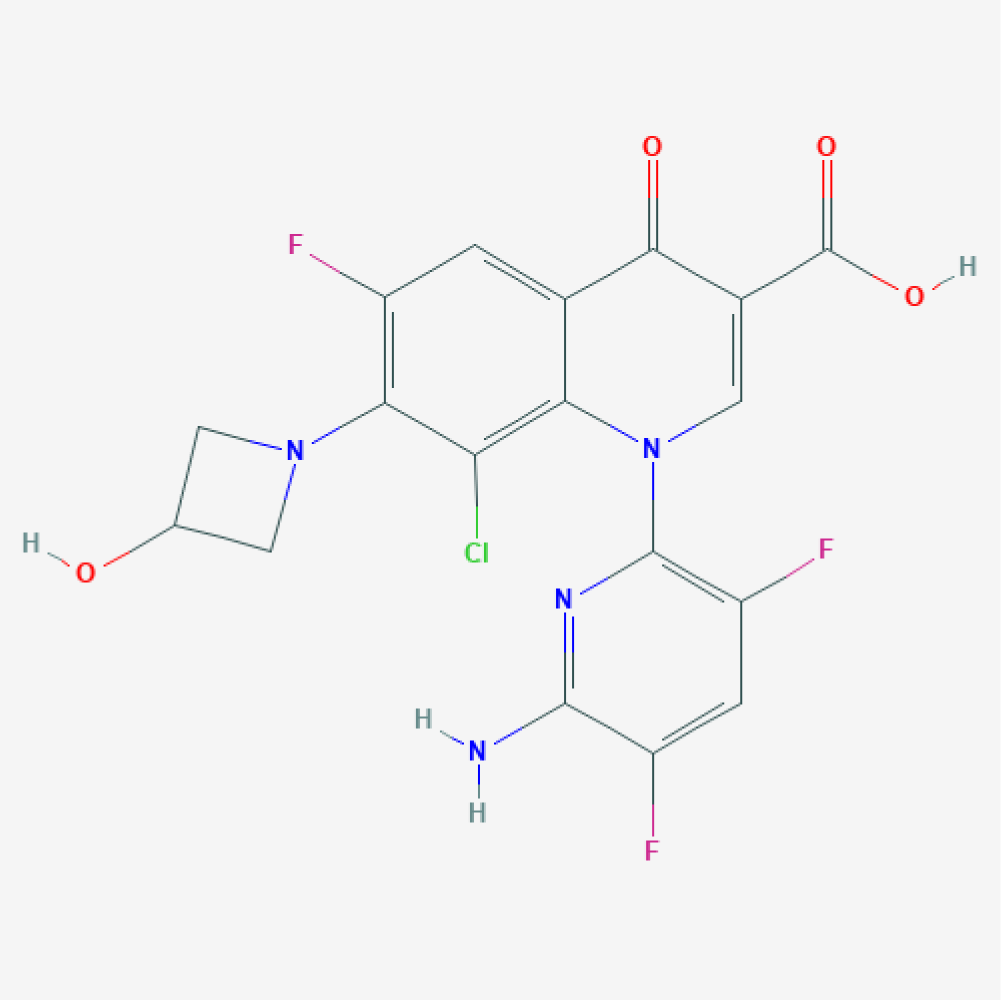

IUPAC Name:
1-(6-amino-3,5-difluoropyridin-2-yl)-8-chloro-6-fluoro-7-(3-hydroxyazetidin-1-yl)-4-oxoquinoline-3-carboxylic acid

9. Ozenoxacin

Chemical name: Ozenoxacin
PubChem CID: 9863827
Molecular Formula: $C_{21}H_{21}N_3O_3$
Molecular Weight: 363.4 g/mol
2D Structure:

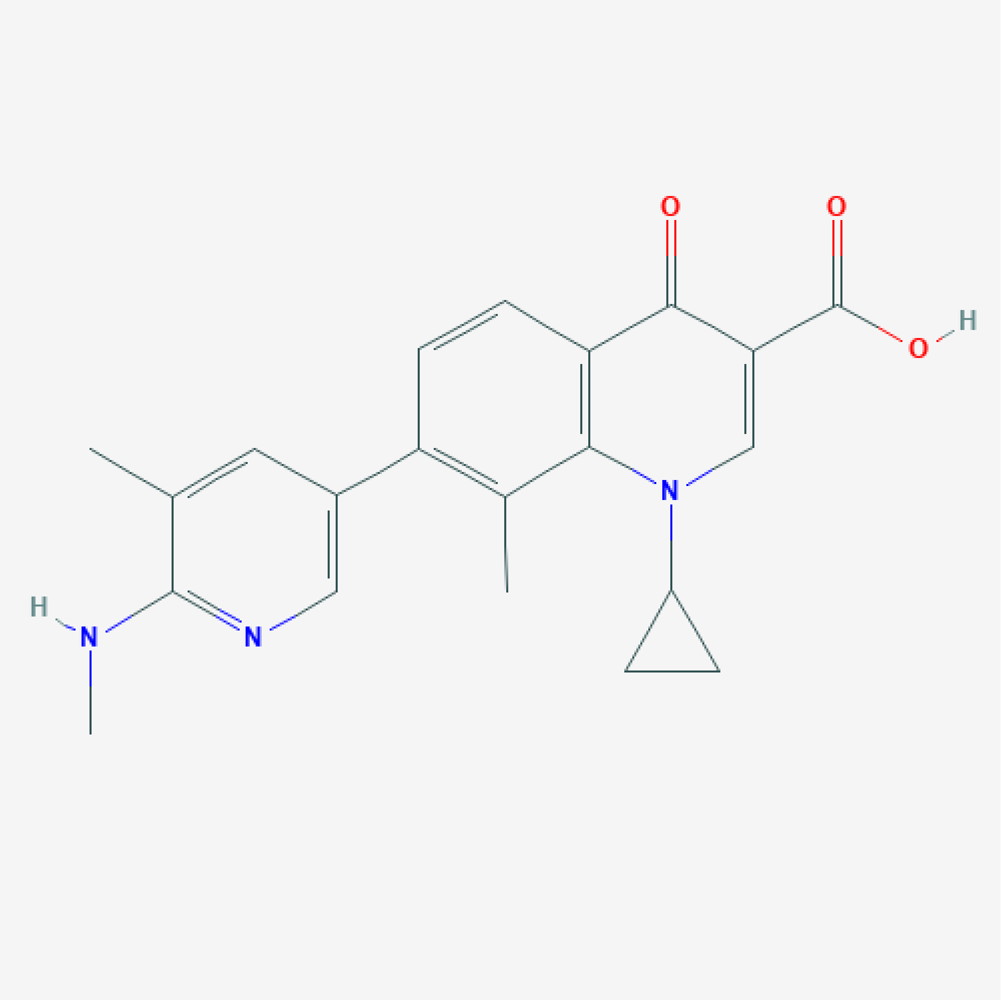

IUPAC Name:
1-cyclopropyl-8-methyl-7-[5-methyl-6-(methylamino)pyridin-3-yl]-4-oxoquinoline-3-carboxylic acid

10. Omadacycline

Chemical name: Omadacycline

PubChem CID: 54697325

Molecular Formula: $C_{29}H_{40}N_4O_7$

Molecular Weight: 556.6 g/mol

2D Structure:

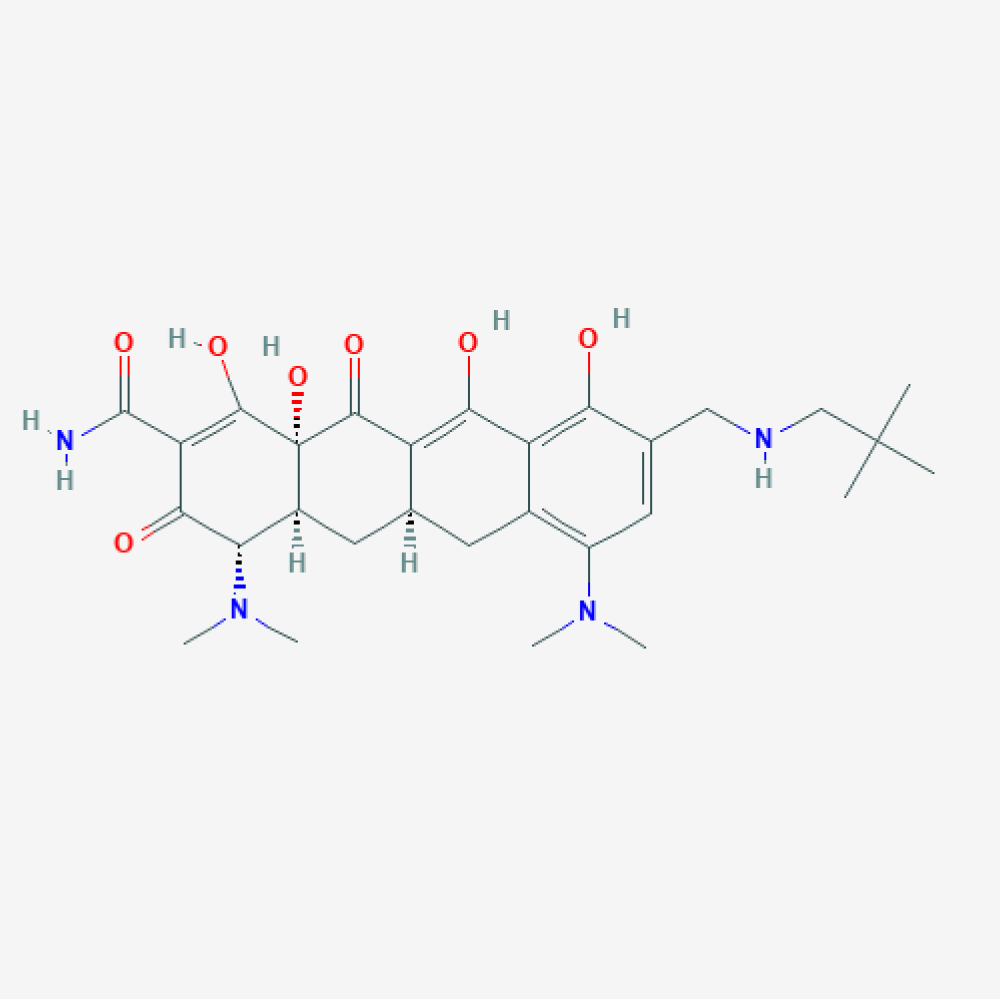

IUPAC Name:

(4*S*,4*aS*,5*aR*,12*aR*)-4,7-bis(dimethylamino)-9-[(2,2-dimethylpropylamino)methyl]-1,10,11,12*a*-tetrahydroxy-3,12-dioxo-4*a*,5,5*a*,6-tetrahydro-4*H*-tetracene-2-carboxamide
